# EGR1 interacts with TBX2 and functions as a tumor suppressor in rhabdomyosarcoma

**DOI:** 10.18632/oncotarget.24726

**Published:** 2018-04-06

**Authors:** Trefa Mohamad, Noor Kazim, Abhinav Adhikari, Judith K. Davie

**Affiliations:** ^1^ Department of Biochemistry and Molecular Biology, Simmons Cancer Institute, Southern Illinois University School of Medicine, Carbondale, IL 62901, USA

**Keywords:** EGR1, TBX2, RMS, apoptosis

## Abstract

EGR1, one of the immediate-early response genes, can function as a tumor suppressor gene or as an oncogene in cancer. The function of EGR1 has not been fully characterized in rhabdomyosarcoma (RMS), a pediatric cancer derived from the muscle linage. We found that EGR1 is downregulated in the alveolar RMS (ARMS) subtype but expressed at levels comparable to normal skeletal muscle in embryonal RMS (ERMS). We found that overexpression of EGR1 in ARMS significantly decreased cell proliferation, mobility, and anchorage-independent growth while also promoting differentiation. We found that EGR1 interacts with TBX2, which we have shown functions as an oncogene in RMS. The interaction inhibits EGR1 dependent gene expression, which includes the cell cycle regulators p21 and PTEN as well as other important cell growth drivers such as NDRG1 and CST6. We also found that EGR1 induced apoptosis by triggering the intrinsic apoptosis pathway. EGR1 also activated two pro-apoptotic factors, BAX and dephosphorylated BAD, which are both located upstream of the caspase cascades in the intrinsic pathway. EGR1 also sensitized RMS cells to chemotherapeutic agents, suggesting that activating EGR1 may improve therapeutic targeting by inducing apoptosis. Our results establish the important role of EGR1 in understanding RMS pathology.

## INTRODUCTION

Rhabdomyosarcoma (RMS) is a malignant mesenchymal origin cancer which is thought to arise from myogenic precursors in the skeletal muscle lineage [1]. Two major subtypes of RMS have been characterized. The embryonal subtype (ERMS) is the most common form of the disease, characterized by the loss of heterozygosity (LOH) at the 11p15 locus [2]. The more aggressive form of RMS is alveolar RMS (ARMS). ARMS is characterized by the chromosomal translocation t (2;13) (q35; q14) or t (1;13) (q36; q14), which generate chimeric transcripts with the 5’ DNA binding site of the paired box protein 3 or 7 (PAX3 or PAX7) fused to the transactivation domain of a forkhead transcription factor (FKHR or FOXO), creating the novel PAX3-FOXO1 or PAX7-FOXO1 oncogenic fusion proteins [3, 4]

Normal skeletal muscle development and differentiation is regulated by expression of group of myogenic regulatory factors (MRFs) including MyoD (MYOD1) and myogenin (MYOG). The MRFs are diagnostic markers for RMS as the MRFs are expressed in almost all RMS tumors. However, RMS tumor cells do not differentiate into skeletal muscle cells and RMS lack factors required for MRF activity [5, 6].

Eukaryotic cell proliferation and differentiation are affected by a number of growth factors and other environmental stimuli. This information is transmitted via signal transduction cascades by the immediate-early response genes, which function as mediators for transmitting extracellular stimuli. The immediate-early response genes include the FOS family (*C-FOS, FRA-1,* and *FOS-B*), the JUN family (*C-JUN, JUNB,* and *JUND*), and the early growth response (EGR) family (*EGR1* and *EGR2*). EGR1 functions as a convergence point for many signaling cascades, as it is stimulated with a variety of environmental signals [7, 8]. EGR1 belongs to the zinc finger transcription factor family and functions in cell growth, differentiation and apoptosis. The zinc finger motif of EGR1 functions as a DNA binding site, whereas the amine terminus confers a transactivation function [9, 10].

EGR1 has been shown to function as both a tumor suppressor and oncogene in cancer. Studies have shown that loss or a low level of EGR1 expression is observed in many human tumors, including breast carcinoma, non-small-cell lung cancer, hematopoietic malignancies, gliomas and sarcomas and that restoration of EGR1 can suppress proliferation and growth of these cells *in vitro* and *in vivo,* suggesting that EGR1 functions as a tumor suppressor [11, 12]. EGR1 has also been shown to function as an oncogene. High levels of EGR1 mRNA expression was seen in prostate cancer tissue compared to normal tissue [13] and blocking EGR1 expression in prostate tumor cell lines showed a decrease in cell proliferation and reversion of the transformed phenotype [14, 15]. Recent studies have also shown that EGR1 silencing has antitumor effects in glioma and colorectal tumor models *in vivo* [16].

In RMS, it has been shown that the chimeric protein PAX3-FOXO1 interacts with and destabilizes EGR1, resulting in a proteasomal degradation of EGR1 which subsequently leads to the loss of functional p57KIP2, a key myogenic regulator which promotes differentiation [17, 18]. The detabilization of EGR1 in ARMS has also been implicatred in the repression of p21 (Hecker *et al*., 2010). In breast cancer, it has been shown that the T-box transcription factor family member, TBX2, interacts with EGR1 and this interaction abrogates EGR1 function as a tumor suppressor and downregulates the transcription of the EGR1 gene-dependent program [19]. We have shown that TBX2 is highly upregulated in RMS and it promotes proliferation and tumorigenesis of RMS cell lines [20]. These results suggested that TBX2 might also block the EGR1 dependent gene program in RMS. We show that EGR1 is expressed in ERMS, but suppressed in ARMS. Restoration of EGR1 in ARMS inhibits proliferation, migration and anchorage independent growth. EGR1 interacts with TBX2 in both ERMS and ARMS and TBX2 antagonizes EGR1 function at a common set of target genes including *NDRG1*, *CST6*, *PTEN* and *CDKN1A* (p21). EGR1 induces apoptosis in ARMS and sensitizes ARMS cells to chemotherapeutic agents. Our novel findings on EGR1 function in RMS highlights the significant role of EGR1 in RMS pathology.

## RESULTS

### EGR1 expression in RMS and normal muscle

**Figure 1:**
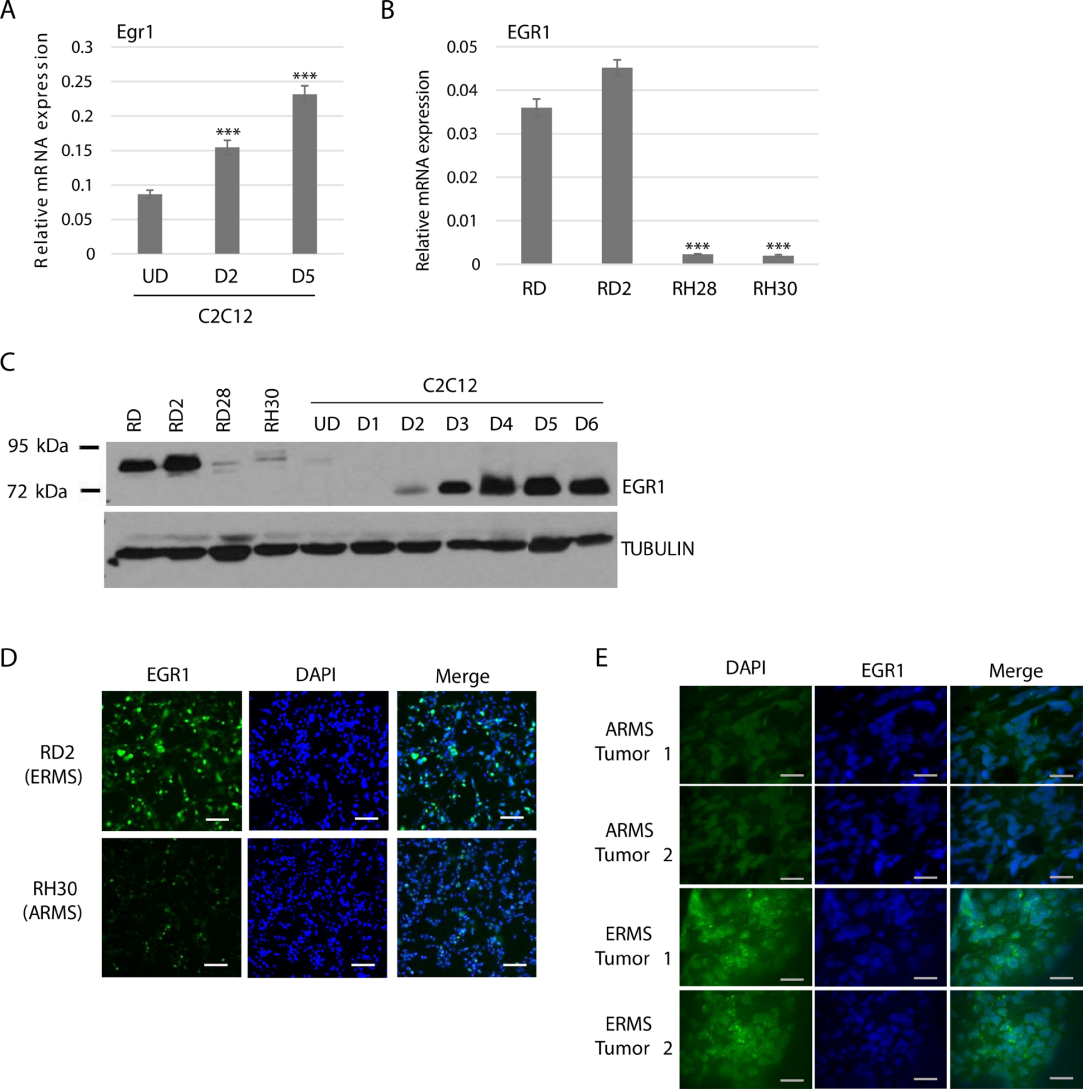
EGR1 is upregulated during muscle differentiation and differentially expressed in RMS (**A**) EGR1 mRNA is up regulated upon C2C12 differentiation. C2C12 cells were assayed for EGR1 expression by qRT-PCR while proliferating (UD) and after 2 days (D2) and 5 days (D5) of differentiation. Error Bars, S.D. and ^***^P > 0.001 vs. UD. (**B**) EGR1 is differentially expressed in RMS. *EGR1* gene expression was assayed in ERMS (RD, RD2) and ARMS (RH28, RH30) cell lines by qRT-PCR. Error bars, S.D. and ^***^P > 0.001 vs. RD. (**C**) EGR1 protein is also differentially expressed. EGR1 protein level in ERMS (RD and RD2), ARMS (RD28 and RH30) and C2C12 cells was assayed by western blot with antibodies against EGR1. (**D**) Immunohistochemistry of RD2 and RH30 cells shows differential EGR1 expression in RMS. Images were taken at 200× magnification and scale bars represent 100 μm (**E**) Human tumors also show differential expression of EGR1 in RMS. Immunohistochemistry was performed on primary human RMS tumor sections with antibodies against EGR1 and DAPI. Images were taken at 200× magnification and scale bars represents 100 μm. Each tumor slide represents an individual tumor.

To understand the function of EGR1 in RMS, we first assayed for the expression of EGR1 in RMS tumor cell lines representing both ERMS and ARMS and a normal myoblast cell line, C2C12, an immortal murine cell line used as a model for normal myogenesis. We assayed for the expression of EGR1 by measuring mRNA and protein expression by both western blot assays and immunohistochemistry. In C2C12 cells, Egr1 mRNA was found to be increased upon differentiation (Figure 1A). In RMS cells, EGR1 mRNA levels were much higher in ERMS cell lines than in ARMS (Figure 1B). EGR1 protein expression was detected with using antibodies against EGR1 which recognize both murine and human proteins, which migrate at different motilities [11]. In agreement with the mRNA results, we found that EGR1 was much more highly expressed in ERMS cell lines compared to ARMS cell lines (Figure 1C). This result is consistent with the earlier work showing the destabilization of EGR1 by the PAX-FOXO1 fusions that characterize ARMS [17]. The higher levels of EGR1 in ERMS cells than in ARMS cells was confirmed by immunohistochemistry in RD2 and RH30 cell lines (Figure 1D). To confirm that these changes could be seen in human tumors, we performed immunohistochemistry on tumor tissue from two independent tumors from both ERMS and ARMS patients and found that ERMS tumors expressed higher EGR1 levels than ARMS tumors (Figure 1E).

### EGR1 inhibits proliferation, cell anchorage-independent growth and migration of ARMS cells

**Figure 2:**
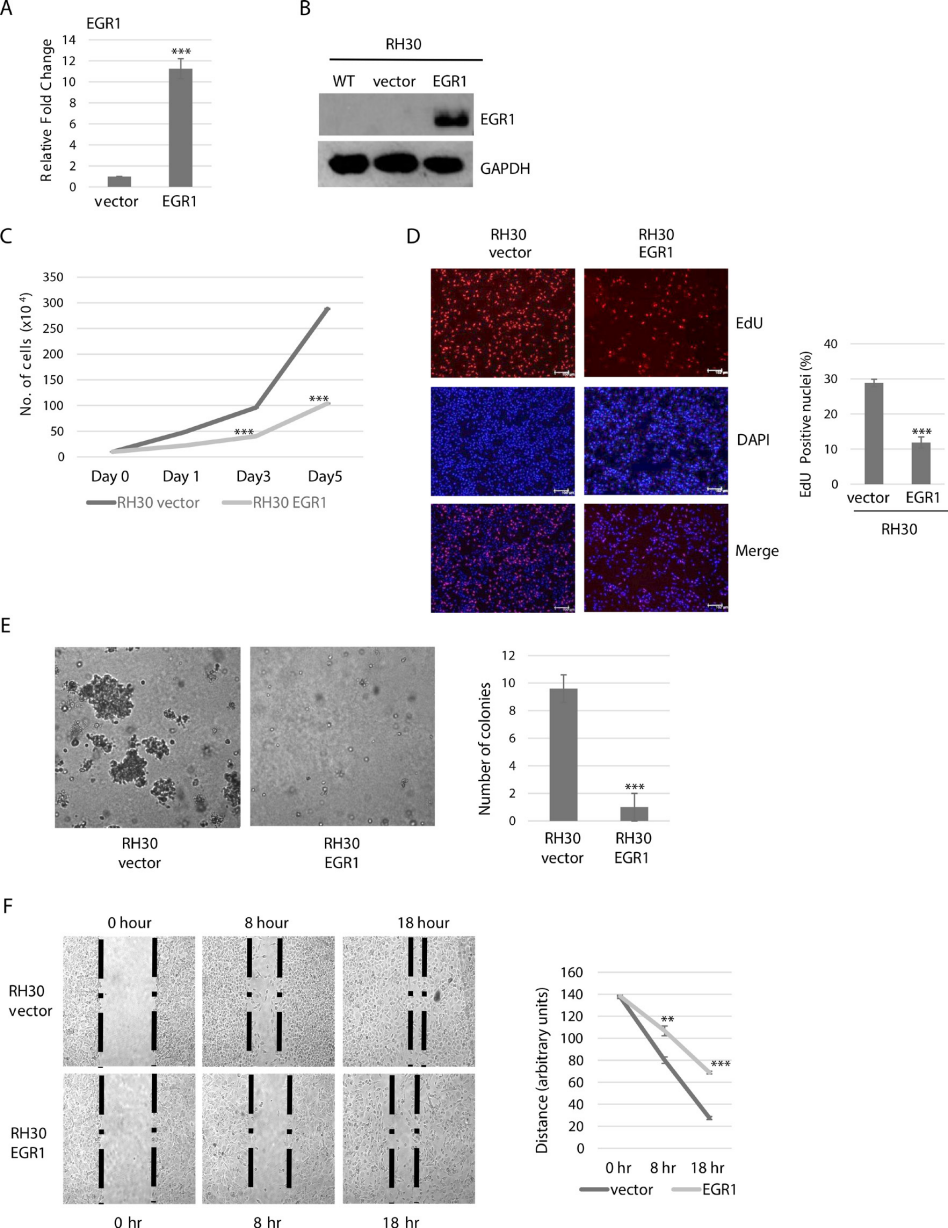
EGR1 overexpression reduces ARMS cell proliferation, anchorage independent growth and migration (**A**) Stable RH30 cell lines overexpressing EGR1 were confirmed by qRT-PCR for mRNA expression (A) and western blot analysis with antibodies against EGR1 (**B**) Error Bars, S.D. and ^***^P > 0.001 vs. vector. (**C**) EGR1 inhibits proliferation of RH30 cells. Cells were seeded at equivalent densities and harvested for cell counts every two days. Error bars, S.D. and ^***^P > 0.001 vs. vector. (**D**) Proliferation assayed by EdU cell proliferation assay. Images are shown on the left and the percentage of EdU positively incorporated nuclei calculated from five independent fields is plotted on the right. Error bars, S.D. and ^***^P > 0.001 vs. vector. (**E**) RH30 cells over expressing EGR1 were used for soft agar colony formation assays. Image were taken at X 100 magnification and scale bars represent 20 μm in left panel. Colony formation was quantitated in right panel by counting in five random fields in each of three independent assays. Error Bars, S.D. and ^***^P > 0.001 vs. vector. (**F**) Scratch assays were performed with RH30 cells stably expressing vector or EGR1. Phase contrast image were taken at the indicated time points of 0 hour, 8 hours, and 18 hours. Images shown in left panel were taken at 100X magnification. Right panel shows quantitation of three independent assays. Error Bars, S.D. and ^**^P > 0.01, ^***^P > 0.001 vs. vector.

To investigate the functional consequences of enhanced EGR1 expression in ARMS cells, we expressed EGR1 ectopically in RH30 cells. Over expression was confirmed at the level of mRNA (Figure 2A), and protein (Figure 2B). As EGR1 has been shown to reduce proliferation in other cancer cell types [12, 21, 22], we assessed RH30 cells stably expressing EGR1 for proliferation and viability and found that the over expression of EGR1 in RH30 cells significantly reduced cell proliferation (Figure 2C). In order to confirm this result, we also quantitated newly synthesized DNA by measuring EdU incorporation and found that RH30 cells stably expressing EGR1 had a reduced number of EdU positive nuclei in comparison to the vector control (Figure 2D).

We also performed a soft agar assay to detect anchorage independent growth. We found that over expression of EGR1 in RH30 highly suppressed anchorage-independent growth, as compared to the vector control cells, which readily formed colonies in soft agar (Figure 2E). EGR1 has also been shown to decrease cell mobility and cell migration in non-small-cell lung cancer [22], thus, we assayed for the effects of EGR1 on cell mobility and migration in RMS cells using a wound healing assay. We found that cell mobility and migration of RH30 cells stably expressing EGR1 was decreased compared with the vector control (Figure 2F).

### EGR1 promotes differentiation

**Figure 3:**
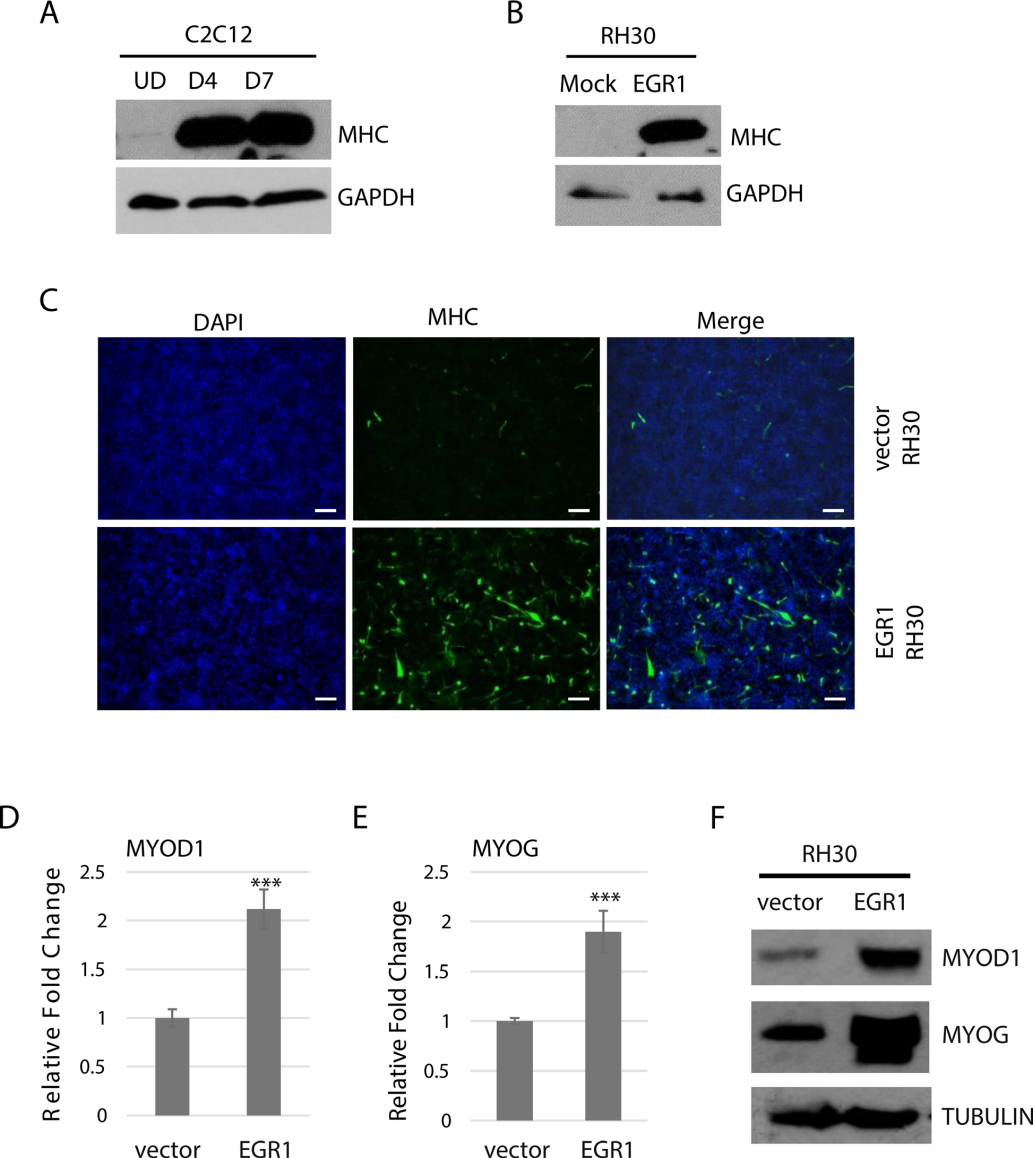
EGR1 promotes differentiation in RMS cells (**A**) MyHC is upregulated upon muscle differentiation. C2C12 cells were assayed for MyHC expression while proliferating (UD) and after 4 days (D4) and 7 days (D7) of differentiation. (**B**) EGR1 over expression induces myosin heavy chain (MHC) expression in RH30 cells. Cells were grown in differentiation medium for 4 days. (**C**) Cells in (B) were immunostained with antibodies against MHC and counterstained with DAPI. (**D**) MyoD and myogenin are upregulated. RNA was extracted from cells as in (B). and gene expression of MYOD1 (D) and MYOG (**E**) assayed by qRT-PCR and protein by western blot analysis (**F**) Error Bars, S.D. and ^***^P > 0.001 vs. vector.

As the data suggested that EGR1 inhibits proliferation, we next asked whether this reduction in proliferation might promote RMS cells toward differentiation. We first assayed for expression of a common marker for skeletal muscle differentiation, myosin heavy chain (MHC). In C2C12 cells, MHC is highly upregulated upon differentiation (Figure 3A). RH30 cells over expressing EGR1 and vector control were subject to differentiation conditions and we found that MHC was highly upregulated in RH30 cells over expressing EGR1 to a level comparable to differentiated myoblasts, while the vector control did not show expression of the MHC protein (Figure 3B). To confirm this result, RH30 cells over expressing EGR1 and vector control were grown in differentiation conditions and immuno-stained with antibodies against MHC. Cells overexpressing EGR1 showed a robust increase in the number of MHC positive cells compared with the control cells (Figure 3C).

We next assayed for the expression of MyoD (MYOD1), which has role in the early differentiation stages of myogenesis, and myogenin (MYOG), which plays an essential role in terminal differentiation and myoblast formation. Gene expression was assayed in RH30 cells over expressing EGR1 and vector control. qRT-PCR data showed an upregulation of mRNA for both MYOD1 (Figure 3D) and MYOG (Figure 3E) and western blot assay results confirmed the upregulation of MYOD1 and MYOG at the protein level (Figure 3F).

### EGR1 interacts with TBX2

**Figure 4:**
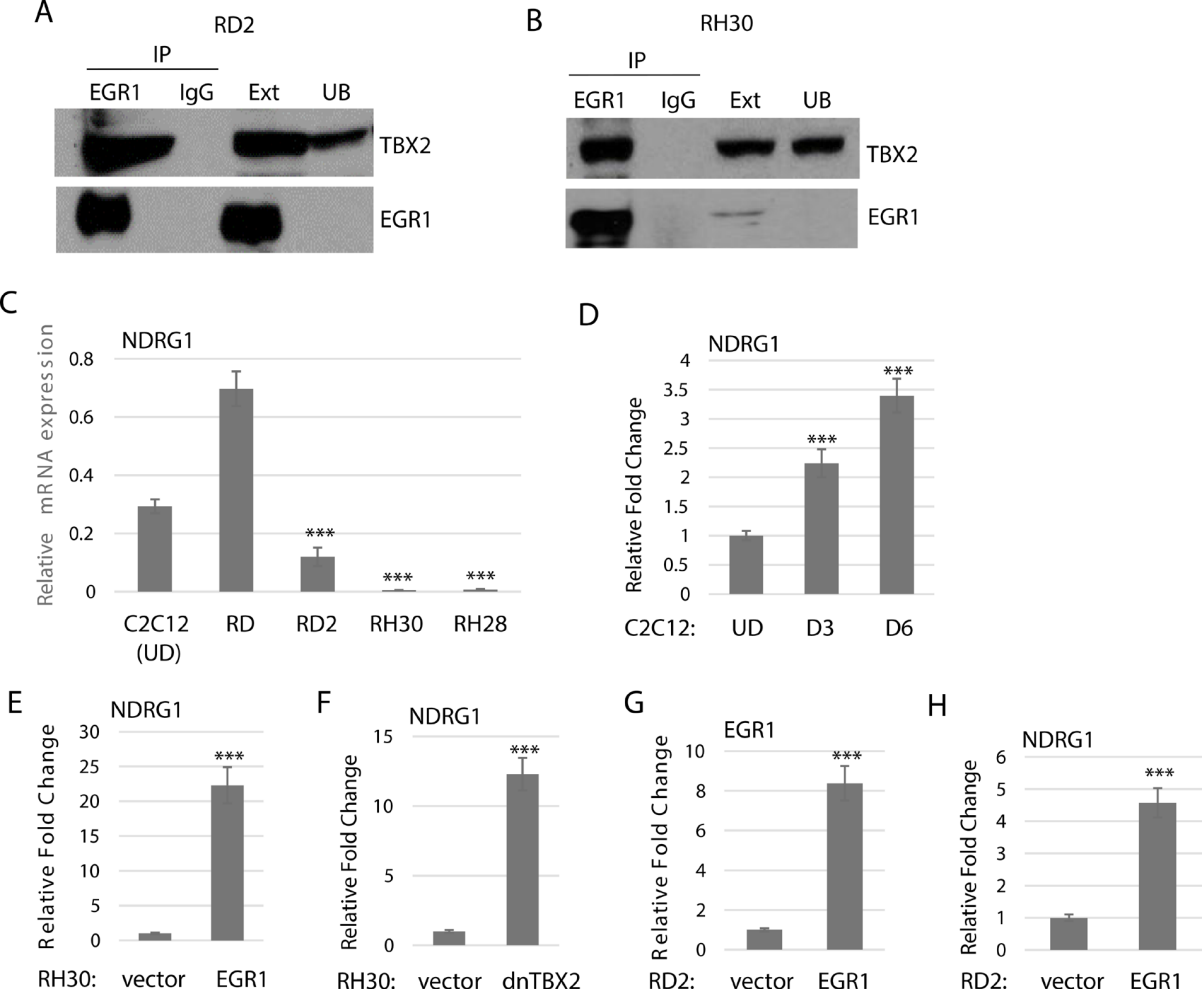
Endogenous EGR1 interacts with TBX2 (**A**) TBX2 interacts with EGR in ERMS. RD2 cell extract was immunoprecipitated with antibodies against EGR1 and probed with antibodies against TBX2 and EGR1. Ext. indicates cell extract and UB indicates unbound supernatant. (**B**) TBX2 interacts with EGR1 in ARMS. RH30 cell extract was immunoprecipitated as in (A). (**C**) NDRG1 expression correlates with EGR1 expression. mRNA expression was quantitated by qRT-PCR in cell lines RD and RD2 (ERMS), RH28 and RH30 (ARMS) and C2C212 undifferentiated cells (UD). Error Bars, S.D. and ^***^P > 0.001 vs. RD. (**D**) NDRG1 in upregulated during normal muscle differentiation. mRNA was quantitated as in (C). for C2C12 cells at the indicated time points. Error Bars, S.D. and ^***^P > 0.001 vs. UD. (**E**) RH30 cells stably overexpressing EGR1 show an upregulation of NDGR1 as assayed by qRT-PCR. Error Bars, S.D. and ^***^P > 0.001 vs. vector. (**F**) Expression of a dominant negative TBX2 (dnTBX2) also upregulates NDRG1 assayed by qRT-PCR as in E. (**G**) EGR1 was transiently expressed in RD2 cells as assayed by qRT-PCR as in (E). (**H**) NDGR1 is upregulated in the cell line described in (G).

Previous studies have shown that EGR1 interacts with TBX2 in breast cancer and represses the function of EGR1 as tumor suppressor [19]. To determine if TBX2 interacted with EGR1 as a potential protein suppressor factor for EGR1 in RMS, we performed a co-immunoprecipitation assay (co-IP). We assayed for the interaction of endogenous EGR1 and TBX2 in RH30 cells, a representative of ARMS, and RD2 cells, a representative of ERMS. Intriguingly, we found that EGR1 interacted with TBX2 in both RD2 (Figure 4A) and RH30 cell lines (Figure 4B).

### Upregulation of TBX2 and EGR1 target genes upon EGR1 over expression

To ask what the functional consequence of the TBX2 and EGR1 interaction was in RMS cells, we examined expression of target genes of either factor, many of which were common to both factors. In breast cancer, the interaction of TBX2 with EGR1 represses the putative breast tumor suppressor, *NDRG1* (N-myc downregulated gene 1), which is implicated in cell differentiation, apoptosis and senescence [19]. We found that NDRG1 expression was higher in ERMS cell lines, RD and RD2, compared to ARMS cell lines, RH28 and RH30 (Figure 4C). In ERMS, which has high levels of both EGR1 and TBX2, NDGR1 was highly expressed in RD but at a much lower level in RD2. We found that NDGR1 was upregulated during normal myogenesis, consistent with its role as a growth control gene (Figure 4D). Ectopic expression of EGR1 in RH30 cells upregulated NDRG1 (Figure 4E), as did ectopic expression of a dominant negative TBX2 (dnTBX2) construct (Figure 4F), which binds DNA but lacks the ability to bind histone deacetylases [23]. To confirm the role of EGR1 on NDRG1 expression in ERMS, we transiently expressed EGR1 in RD2 cells and found that EGR1 expression increased as anticipated (Figure 4G) and NDRG1 expression also increased (Figure 4H).

**Figure 5:**
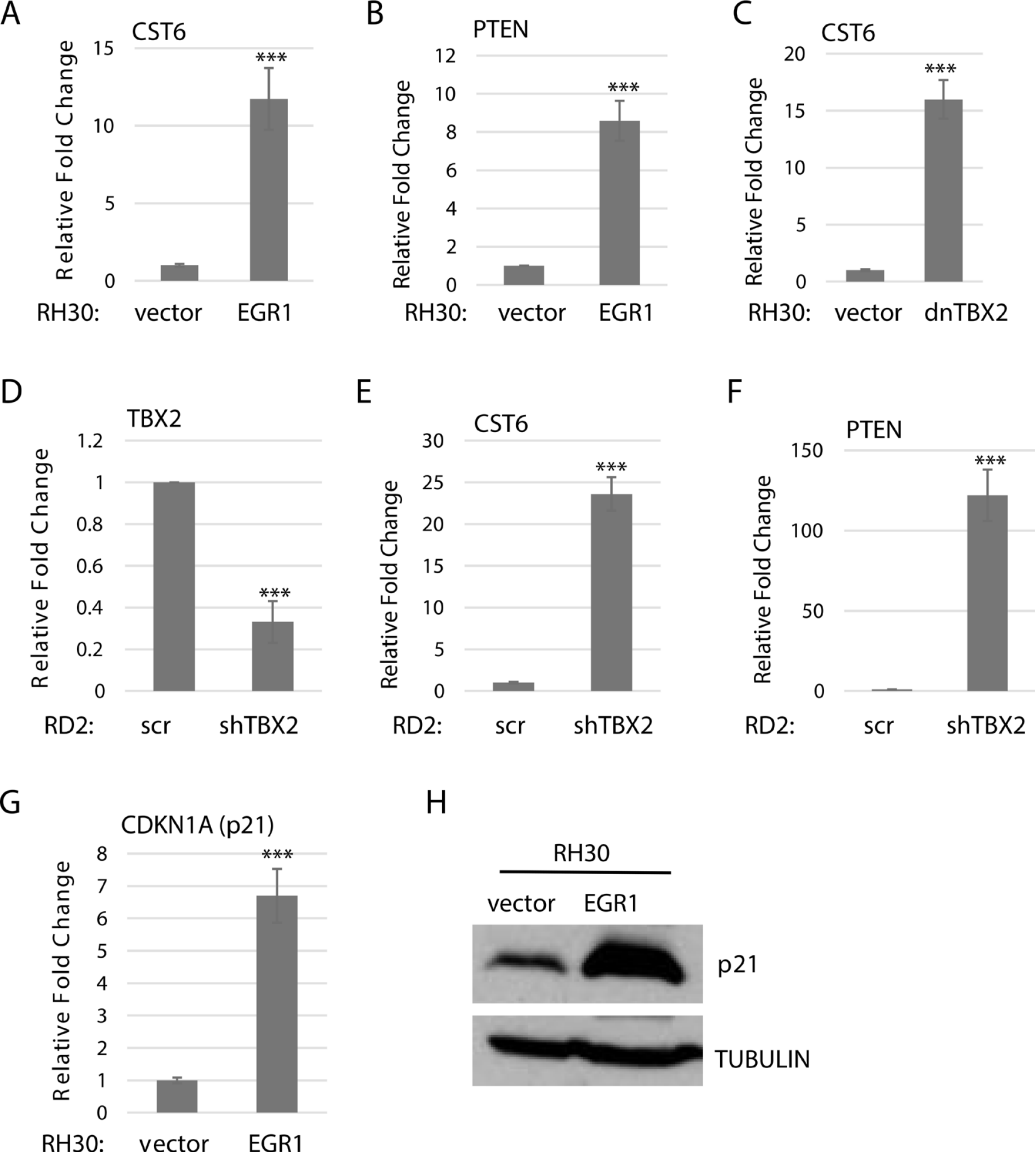
TBX2 antagonizes EGR1 on target gene expression RH30 cells stably overexpressing EGR1 upregulate CST6 (**A**) and PTEN (**B**) as assayed by qRT-PCR. Error Bars, S.D. and ^***^P > 0.001 vs. vector. (**C**) RH30 cells transfected with dnTBX2 upregulate CST6 as assayed by qRT-PCR as in A. (**D**) RD2 cells transfected with a shRNA against TBX2 were assayed for TBX2 depletion by qRT-PCR. Cells from (D). were assayed for CST6 (**E**) and PTEN (**F**) by qRT-PCR as in (A). (F) CDKN1A (p21) is upregulated in RH30 cells stably overexpressing EGR1 at the mRNA (**G**) and protein (**H**) level.

We then expanded this analysis to additional targets including cystatin 6 (CST6), a cysteine protease inhibitor which functions as a tumor suppressor and has been identified as a repression target of TBX2 through a mechanism involving EGR1 [24]. We found that CST6 mRNA was upregulated in RH30 cells overexpressing EGR1 (Figure 5A). We also examined the tumor suppressor and pro-apoptotic gene, phosphatase and tension homolog detected on chromosome 10 (PTEN), which is known to be activated by EGR1 and repressed by TBX2 in many cancer types [25, 26]. We have shown that PTEN is repressed in RMS by TBX2 [27] and here we found a significant up-regulation of PTEN mRNA in RH30 cells overexpressing EGR1 (Figure 5B). In RH30 cells expressing dnTBX2, we also found an upregulation of CST6 (Figure 5C). In ERMS, transient depletion of TBX2 (Figure 5D) upregulated both CST6 (Figure 5E) and PTEN (Figure 5F). Finally, we examined the effect of EGR1 on a well-established target of TBX2, p21 [28], that we have previously shown to be a direct target of TBX2 in RMS [20]. We found that RH30 cells overexpressing EGR1 upregulated p21 at both the mRNA (Figure 5G) and protein level (Figure 5H). Taken together, the results strongly suggest that TBX2 antagonizes the function of EGR1 in RMS on a common set of gene targets.

### EGR1 promotes apoptosis

**Figure 6:**
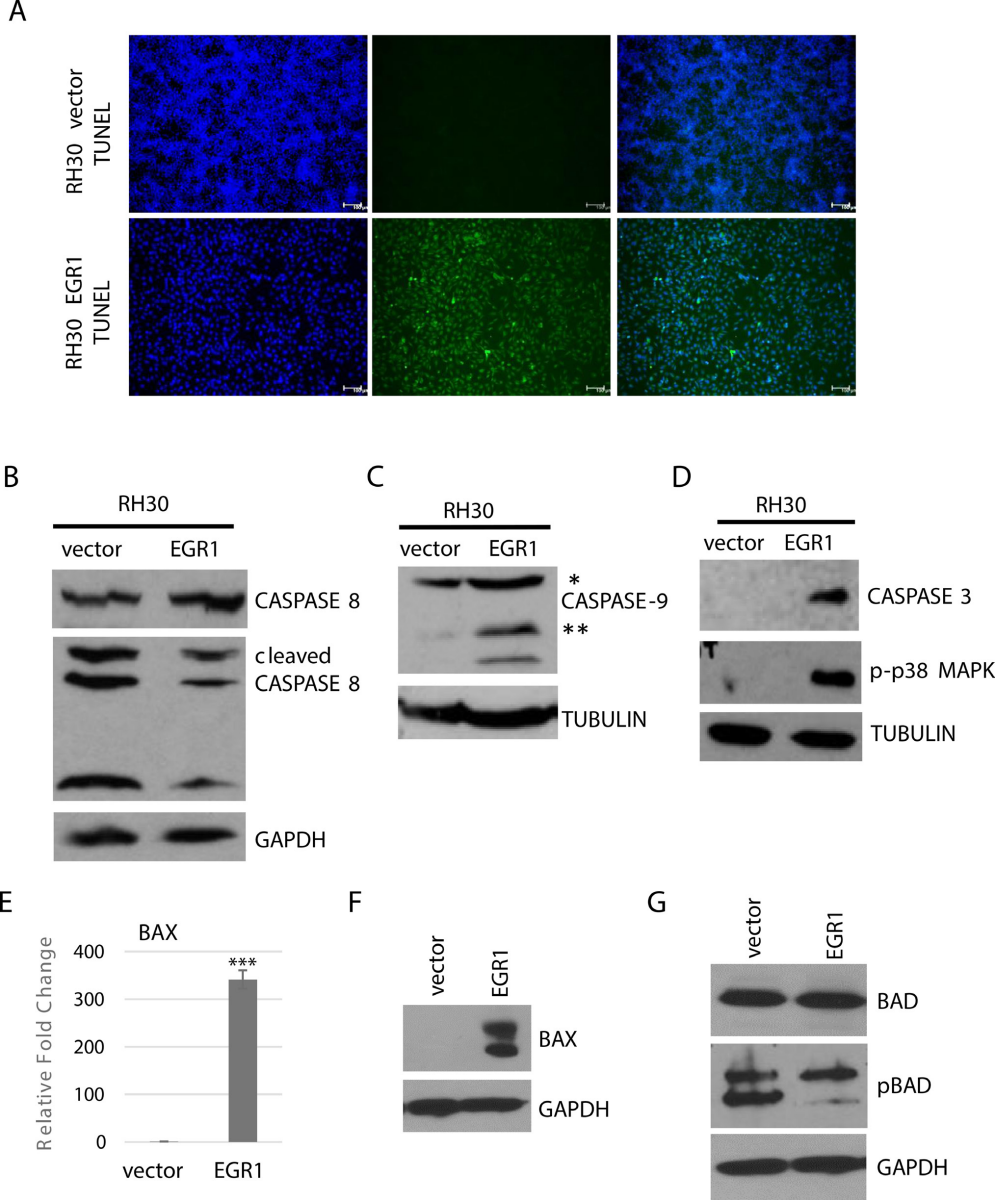
EGR1 over expression induces intrinsic cell apoptosis (**A**) RH30 cells over expressing EGR1 or vector control were subject to differentiation conditions for 4 days and apoptosis was detected by TUNEL assay. (**B**) Caspase 8 cleavage is not affected by EGR1. Cells as described in A were assayed by western blot analysis. (**C**) Caspase 9 cleavage is induced by EGR1, assayed as in (B). Single asterisk indicates full length protein; double asterisks indicate cleaved products. (**D**) Caspase 3 and p-p38 are induced by EGR1, assayed as in B. BAX1 is upregulated by EGR as assayed by qRT-PCR (**E**) and western blot assay (**F**). Error Bars, S.D. and ^***^P > 0.001 vs. vector (**G**) Phosphorylated BAD (pBAD) is downregulated in response to EGR1.

During the course of our study, we noted that when RH30 cells expressing EGR1 were subject to differentiation conditions, many of the cells showed a rounded appearance, which could indicate apoptosis. In addition, EGR1 has been indicated in the induction of apoptosis in a variety of cancers [16, 29, 30]. Thus, we assayed for apoptosis in these cells by TUNEL assays, which detects chromosome fragmentation in the later stages of apoptosis. We found that TUNEL+ cells could be readily observed in RH30 cells overexpressing EGR1 and were not observed in RH30 cells expressing vector (Figure 6A).

To understand the apoptotic cascade initiated in these cells, we examined caspase cleavage. Apoptosis can be trigged through intrinsic or extrinsic pathways which signal through distinct initiator caspases [31]. Caspase-8 activation is required for the extrinsic pathway and we found no changes in caspase 8 expression or cleavage patterns (Figure 6B). Caspase-9 activation is required for the intrinsic pathway and enhanced expression and cleavage of caspase-9 was seen in RH30 cells overexpressing EGR1 (Figure 6C). Activation of the initiator caspases converge on the activation of executioner caspases such as caspase-3, and expression of caspase-3 was higher in RH30 cells overexpressing EGR1 compared to vector control (Figure 6D). p38 MAPK is closely associated with the initiation of apoptotic events and we found robust phosphorylation of p38 MAPK in RH30 cells overexpressing EGR1 while the vector control showed no activation of p38 (Figure 6D).

The *BAX* gene, a member of the Bcl-2 apoptotic protein family with pro-apoptotic activity, has previously been shown to be a direct target of EGR1, which subsequently led to apoptosis induction in pancreatic cancer cells [32]. We found that BAX mRNA expression was highly upregulated upon the over expression of EGR1 in RH30 cells following differentiation conditions (Figure 6E). The expression of BAX was confirmed at the protein level as well (Figure 6F). We also examined the expression and serine dephosphorylation of BAD (Bcl-2 antagonist of cell death). When phosphorylated, BAD associates with 14-3-3 and is sequestered in the cytosol but when unphosphorylated, BAD translocates to the mitochondria to release cytochrome 3 [33]. BAD can also heterodimerize with the anti-apoptotic Bcl-2 family members Bcl-X1 and Bcl-2, thus neutralizing their protective effect [34]. We found that while total BAD was unchanged, phosphorylated BAD was highly reduced in RH30 stably expressing EGR1 compared to the vector control (Figure 6G)

### EGR1 sensitizes cells to chemotherapeutic drugs

**Figure 7:**
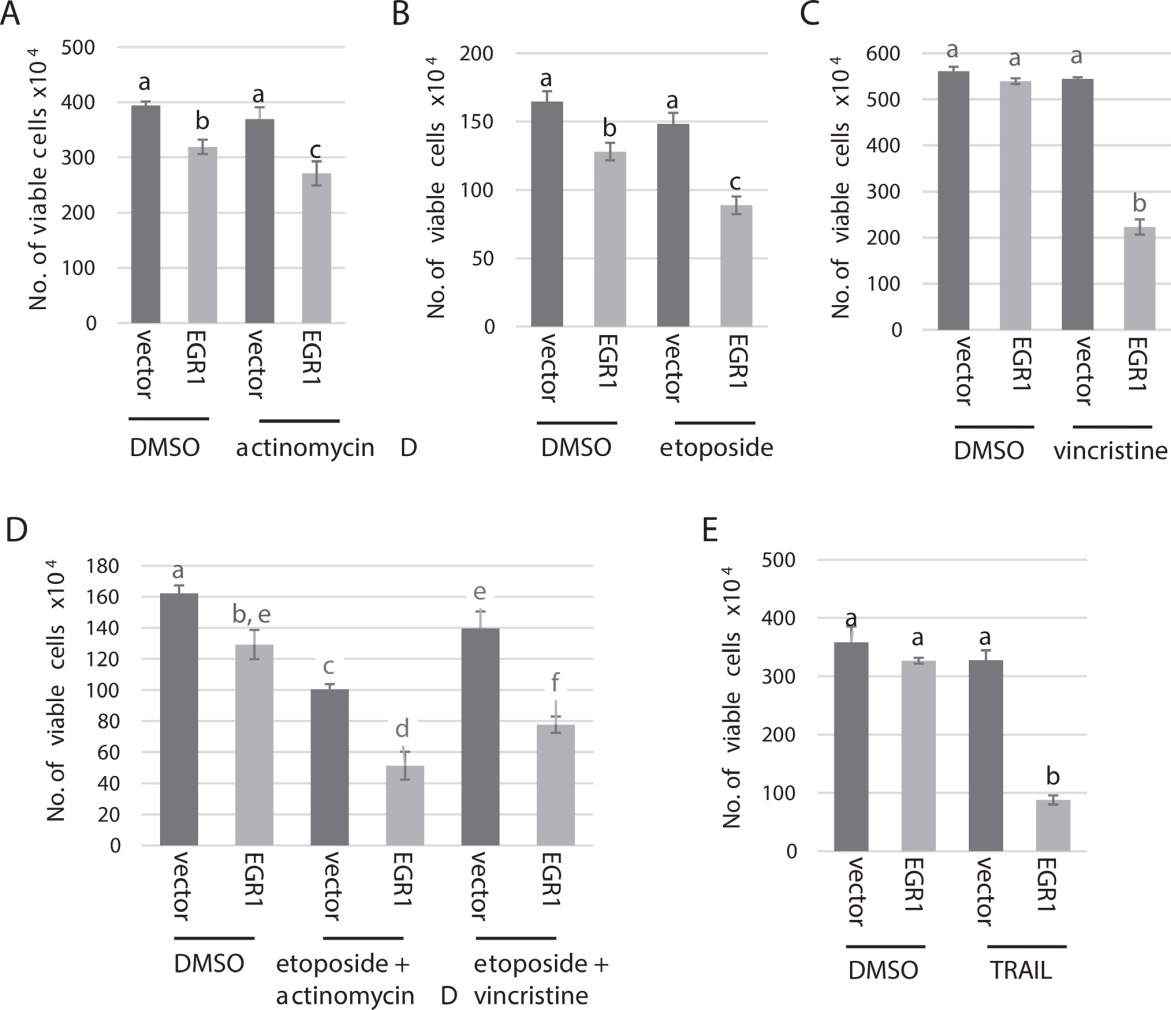
EGR1 sensitizes RH30 cells to chemotherapeutic drugs RH30 cells over expressing EGR1 or vector control were treated with actinomycin D or vehicle control (DMSO). (**B**) Cells in (**A**) were treated with etoposide or vehicle control (DMSO). (**C**) Cells in A. were treated with vincristine or vehicle control (DMSO). (**D**) Cells in A. were treated with a combination of etoposide and actinomycin D, etoposide and vincristine or vehicle control (DMSO). (**E**) Cells in A. were treated with TRAIL. All data are plotted as means with SD and analyzed with one-way ANOVA followed by Tukey's HSD (honest significant difference) post-hoc test. Means not sharing same letter are statistically significant (*p* < 0.01).

We next asked whether over expression of EGR1 can sensitize RMS cells to chemotherapeutic drugs. We tested the response to several chemotherapeutic agents currently used to treat RMS including actinomycin D, which is antitumor drug that prevents nucleic acid synthesis; vincristine, an alkaloid agent that inhibits mitosis in M-phase through interfering with the microtubular spindle proteins which are essential for cell division; and etoposide, which works by blocking topoisomerase 2 [35]. We found that over expression of EGR1 significantly sensitized RH30 cells to actinomycin D (Figure 7A), etoposide (Figure 7B) and vincristine (Figure 7C), with the most dramatic effect seen for vincristine. We also treated these cells with chemotherapeutic drug combinations and found that EGR1 also sensitized cells to combined drug treatments (Figure 7D). Finally, we treated the EGR1 over expressing RH30 cell line with TRAIL and found that these cells were highly sensitized to TRAIL compared to the vector control (Figure 7E). These results suggest that restoration of EGR1 expression in RMS may enhance the response of tumor cells to chemotherapeutic drugs.

## DISCUSSION

In this work, we show that EGR1 functions as a tumor suppressor in RMS. Over expression of EGR1 in ARMS led to a significant growth inhibition, consistent with results in many other cancers. TBX2 interacts with EGR1 and antagonizes the function of EGR1 in both ERMS and ARMS. The balance between these two critical cell growth regulators in RMS has a profound impact on tumor cell growth and apoptosis through co-regulation of essential cell growth and cell cycle control genes such as *CDKN1A* (p21), *PTEN*, *NDRG1* and *CST6*.

The most effective function of a tumor suppressor in cancer cells is to activate and induce apoptosis and we find that EGR1 initiates apoptosis though the internal pathway in ARMS cells as detected by activation of caspase 9. EGR1 has been previously indicated in the induction of apoptosis in a variety of cancers [16, 29, 30, 36]. The EGR1 induced apoptotic response we observed also involved the phosphorylation of p38 MAPK. p38 MAPK is closely associated with the initiation of apoptotic events in many types of cells and several of the anti-tumor compounds which induce apoptosis in cancer cells do so by activating and phosphorylating the p38 MAPK/JNK signaling pathway [37, 38].

We found that exposure to serum starvation (differentiation conditions) is essential to initiate the apoptotic response induced by EGR1 and we currently do not understand the mechanistic basis for this effect. EGR1 is an early growth response gene that transits external signals, so it is likely that serum starvation is altering a signaling pathway that impacts EGR. In lung pathogenesis, it has been found that two signaling pathways required for tissue remodeling depend on EGR1 initiated apoptotic cascades. IL-13 signals through EGR1, which is required to initiate the IL-13 induced apoptotic cascade including reduced levels of caspase 3, 7, 9 and Bax (Cho *et al*., 2006) and TGF-beta also promotes apotosis though EGR1 [39].

Expression of a pro-apoptotic Bcl-2 member, BAX, was found to be robustly induced by EGR1. EGR1 has been shown to act as a pro-apoptotic factor in pancreatic cancer cells through direct induction of BAX [32, 40]. The induction of BAX we observe may be due to the direct activation of BAX by EGR1, but BAX is also known to be induced by the phosphorylation of p38, which we also see is induced by EGR1 expression. Our data suggest that EGR1 functions as a pro-apoptotic factor in RMS through its ability to activate these downstream pro-apoptotic factors. Previous work has also shown that expression of the proto-oncogene *BCL-2* is inversely correlated with EGR1 expression, suggesting that EGR1 may also be a negative regulator of the anti-apoptotic effect of BCL-2 [41].

The prognosis of childhood rhabdomyosarcoma is still poor. Currently, treatment approaches of RMS involve a combination therapy of chemotherapeutic agents, complete surgical resection and radiation therapy [42]. Our results suggest that the low level of EGR1 in ARMS and the antagonistic function of TBX2 on EGR1 in ERMS contributes to chemotherapeutic resistance in RMS. Low levels of EGR1 have been implicated in chemotherapeutic resistance in two other cancers. In triple negative breast cancers (TNBCs), the Y-box binding protein (YB-1) promotes paclitaxel (PTX) resistance by reducing EGR1 levels and cells with lower levels of EGR1 were more resistant to PTX [43]. PTX acts by stabilizing microtubules, thus, cells are unable to proceed through mitosis and are eliminated by apoptosis. Ovarian cancer cell line studies have also demonstrated that low levels of EGR1 are associated with PTX resistance [44]. Here, we found that restoration of EGR1 sensitizes ARMS cells in their response to chemotherapeutic drugs commonly used to treat RMS patients, including actinomycin D, vincristine and etoposide. The results with vincristine were particularly striking as vincristine serves as the backbone for the majority of therapeutic regimes for RMS [45]. The standard chemotherapeutic treatment for RMS are combination therapies which include the frequently employed vincristine, actinomycin D and cyclophosphamide (VAC) protocol [35] and our data show that EGR1 sensitizes RMS cells to combined drug treatments. We also found that EGR1 overexpression in ARMS cells highly sensitized ARMS cells to TRAIL, consistent with studies in acute myelogenous leukemia (AML) where EGR1 over expression was shown to sensitize cells to TRAIL-induced apoptosis [46].

Triggering the apoptosis machinery is considered to be a main function of chemotherapeutic agents in treating all cancer types and defects in apoptosis pathways contribute to the development of a drug resistant tumor [47]. We show here that the loss of EGR1 in ARMS contributes to the resistance of RMS cells to apoptosis. Our results suggest that using EGR1 as a therapeutic target in RMS cells might be a new treatment approach to improve the sensitivity of RMS to chemotherapeutic drugs. It will an important future direction of this work to determine if reactivation of EGR1 in tumor cells can sensitize tumors to chemotherapy *in vivo*. While EGR1 is destabilized by PAX-FOXO1 in ARMS, we show that enhanced expression can overcome this effect and we are currently investigating other regulatory mechanisms that inhibit the expression of EGR1 in RMS. Our data suggest that EGR1 and TBX2 are critical mediators of cell growth and apoptosis in RMS. Altering the balance of these two opposing factors can induce apoptosis and has the potential to improve the therapeutic targeting of RMS.

## MATERIALS AND METHODS

### Cell culture

RD (ATCC), RD2, RD28, SJRH30 (RH30) (ATCC) were grown in Dulbecco's modified Eagle Medium (DMEM) supplemented with 10% fetal bovine serum (Hyclone) in a humidified CO^2^ incubator at 37° C according to standard protocols [48]. C2C12 (ATCC) myoblast proliferation was maintained using DMEM media supplemented with 10% fetal bovine serum. To induce myotube differentiation, C2C12 cells were grown to 70% confluence and the media was switched to DMEM supplemented with 2% horse serum (Hyclone). C2C12 cells were grown in differentiation medium for number of days as indicated in each experiment. RD2 and RH28 were obtained from Dr. Denis Guttridge (Ohio State University). All cell lines were authenticated by Bio-Synthesis (Lewisville, TX) using STR analysis on September 14, 2011.

### Plasmids

Expression construct pcDNA3.1/Zeo (+)/hFlag-EGR1 was kindly provided by Dr. Dan A. Dixon (University of Kansas Medical Center). The dnTBX2 construct (1-301aa) was kindly provided by Colin Goding (University of Oxford). The shRNA constructs against TBX2 were previously described [20].

### Quantitative real time PCR (qPCR)

Trizol (Life Technologies, Carlsbad, CA) was used for RNA extraction from cells. Extracted RNA was treated with DNase (Promega, Madison, WI) and 2 μg of total RNA was reverse transcribed with MultiScribe MuLV reverse transcriptase (Life Technologies). 40 ng cDNA was used for quantitative polymerase chain reaction (qPCR) amplification (Life Technologies, Carlsbad, CA) with SYBR green PCR master mix (Life Technologies, Carlsbad, CA). Negative controls included no RT samples where no reverse transcriptase was added for each RNA sample. All quantitative RT-PCR (qRT-PCR) was performed in triplicate and three independent RNA samples were assayed for each time point. qRT-PCR data were calculated using the comparative Ct method (Life Technologies). Standard deviations from the mean of the [Δ] Ct values were calculated from three independent RNA samples. Primers used are listed in Supplementary Table 1. Where possible, intron spanning primers were used. qRT-PCR gene expression data are shown using two formats. For measurements of relative gene expression (Relative Fold Change), a fold change was calculated for each sample pair and then normalized to the fold change observed at *HPRT*. For relative measurements of mRNA expression levels (Relative mRNA Expression), gene expression levels were quantitated using a calibration curve based on known dilutions of concentrated cDNA. Each mRNA value was normalized to that of *HPRT*. Samples were also compared against 18S to confirm the normalization.

### Western blot

Cell extracts were made by lysing phosphate-buffered saline (PBS) washed cell pellets in RIPA buffer supplemented with protease inhibitors (Complete, Roche Diagnostics, Indianapolis, IN) and clear lysates obtained by centrifugation. Protein concentration was determined by Bradford's assay (Bio-Rad) and 50 μg of protein was loaded for each well of sodium dodecyl sulfate polyacrylamide gel electrophoresis (SDS-PAGE). Resolved proteins were then transferred onto a PVDF membrane using a tank blotter (Bio-Rad). Membranes were blocked with 5% milk in 1X Tris-buffered saline plus Tween 20 (TBST) and followed by incubation with primary antibody overnight at 4° C. After washing membranes with 1X TBST, membranes were incubated with the corresponding secondary antibody, washed with 1X TBST and incubated with chemiluminescent substrate according to the manufacture's protocol (SuperSignal, Pierce) and visualized by autoradiography.

The antibodies used include anti-EGR1 (T.160.5) (Thermo Scientific), anti-TBX2 (C-17 Santa Cruz Biotechnology), anti-GAPDH (Millipore), anti-caspase 9 (9502, Cell Signaling), anti-caspase 3 (Cell Signaling), anti-BAD (9292S, Cell Signaling), anti-p-BAD (Ser136) (4366P, Cell Signaling), anti-caspase 8 (1C12) (9746S, Cell Signaling), anti-cleaved Caspase 8 (Asp391) (18C8) (9496s, Cell Signaling), anti-tubulin (E7, Developmental Studies Hybridoma Bank (DSHB)), anti-MyoD (5.8A Santa Cruz Biotechnology), anti-myogenin (F5D, DSHB), anti-p21 (2947, Cell Signaling), anti-p-p38 MAPK (4511, Cell Signaling), anti-myosin heavy chain (MF-20, DSHB) and anti-BAX (2772S, Cell Signaling).

### Cell transfections

Cells were transfected with calcium phosphate according to standard protocols or Turbofect transfection reagent (Thermo Scientific). Transient transfections were harvested 48 hours post transfection. Stable cell lines were made by transfecting cells with linearized plasmids and selecting for drug resistant colonies. Individual clones were isolated and propagated.

### Co-immunopreciptation

150–300 μg of whole cell extracts made in RIPA buffer were used for each immunoprecipitation and incubated overnight with 1 μg of antibody at 4° C. Antibody/antigen complexes were pulled down with protein A beads (Life Technologies), and western blot was used to analyze the candidate interacting protein in each complex. The blot was probed with antibodies against the reciprocal factor for each immunoprecipitation to detect the co-immunoprecipitation, and with antibodies used for the immunoprecipitation to confirm that the IP was successful. All immunoprecipitations were performed at least twice to confirm the results.

### EdU staining assay

Cells were assayed using a Click-iT EdU Alexa Flour 488 Imaging Kit according to the manufacture's protocol (Life Technologies). EdU positive nuclei were counted in five random fields on microscopic images taken at 200 X magnification using a Leica Microscope.

### Immunohistochemistry

Cells were grown on coverslips before fixation with 4% formaldehyde. Cells were permeabilized and blocked by incubating with goat serum and 1.0% NP-40 for 1 hour. Primary antibodies were added and incubated at room temperature for 2 hours. Coverslips were washed three times with 1X PBS, then secondary antibodies conjugated with Alexa Flour-488 goat anti-mouse or anti-rabbit antibodies (1:500, Life Technologies) were incubated at room temperature for 2 hours and protected from light. Cell nuclei were stained by incubating with DAPI (1 μM, Life Technologies) for 5 minutes at room temperature.

Primary human RMS tumor samples on slides and accompanying pathology reports with no patient information except age and sex were obtained from the Nationwide Children's Hospital Biopathology Center, Columbus, Ohio. Samples were fixed by 3% H_2_O_2_ in methanol for 20 minutes at room temperature, washed in PBS and blocked by 0.1% Triton X-100 TBS solution supplemented with 1.5% normal goat serum overnight at 4° C. Primary antibodies were incubated for 2 hours at room temperature, and then washed with 0.1% Triton X-100 TBS before adding the corresponding secondary antibodies Alexa Fluor-488 goat anti-mouse (1:500, Invitrogen) for incubation for 1 hour at room temperature.

Primary antibodies used in immunohistochemistry included anti-EGR1, (T.160.5 Thermo Fisher) and anti-myosin heavy chain (MF20, Developmental Studies Hybridoma Bank),

### Proliferation assay

Cells were seeded in 6-well plates of 4 × 10^4^ cells per well and on the indicated days they were harvested for counting by hemocytometer. Cell viability was determined by trypan blue staining. Cell counting was performed in duplicate for three blinded biological replicates.

### Soft agar assay

Soft agar assays were carried out in 60 mm dishes in which 2 ml of 0.7% Noble agar (USB) in 1X DMEM with 10% FBS was overlaid with 2 ml of 0.35% agar in 1X DMEM with 10% FBS containing the cells. RH30-pcDNA3.1 (vector) and RH30-EGR1 cells were grown to 100% confluence, trypsinized, and dispersed. Cells of each clone (3 × 10^5^) were plated in triplicate. 1 ml of culture medium was added to the top of each plate every 5 days and cells were grown at 37° C for 30 days. The plates were stained with 1 ml of 0.05% Crystal Violet (Fisher) for >1 hour and colonies were counted using a dissecting microscope.

### Scratch assay

Cell mobility was assayed by scraping a straight line with a 10 μl pipet tip on a monolayer of cells grown to 100% confluency. Cell debris was removed by washing. To obtain the same field during the image acquisition, marks were created near the scratch line. The plate was placed in a CO_2_ incubator at 37° C for the indicated hours.

### Drug treatment

Cells were seeded in triplicate in a 6-well plate with 30×10^4^ cells per well. After recovery for 24 hours, the cells were treated with 10 nM vincristine for 72 hours (Cayman Chemical), 10 nM actinomycin D for 48 hours (Cayman Chemical), and with 50 μM etoposide for 24 hours (Acros Organics) and with 0.5 mg/ml TRAIL (EMD Millipore) for 12 hours. For multidrug treatment, combination of etoposide (50 μM) and vincristine (10 nM), or etoposide (50 μM) and actinomycin D (10 nM) were added to the cells and incubated for 24 hours. Cell viability was determined using trypan blue staining, and cell number was counted in triplicate. All assays were performed at least three times to confirm results.

### Statistics

Data are presented as means ± standard deviation (SD). Statistical comparisons were performed using unpaired two-tailed Student's *t* tests or one-way ANOVA followed by Tukey's HSD (honest significant difference) post-hoc test. Probability value of < 0.05 was taken to indicate significance.

## SUPPLEMENTARY MATERIALS AND TABLES


