# Supplementary material for: EGR1 interacts with TBX2 and functions as a tumor suppressor in rhabdomyosarcoma
Source: Oncotarget. 2018 Apr 6;9(26):18084–98. doi: 10.18632/oncotarget.24726 (PMC5915059; doi:10.18632/oncotarget.24726)
Supplement: Supplementary file 1 [file oncotarget-09-18084-s001.pdf]

EGR1 interacts with TBX2 and functions as a tumor suppressor in rhabdomyosarcoma

SUPPLEMENTARY MATERIALS

Supplementary Table 1: Primers used for quantitative real time PCR (qRT-PCR)

18S rRNA	F 5′ CGCCGCTAGAGGTGAAATTCT 3′
	R 5′ CGAACCTCCGACTTTCGTTCT 3′
HPRT1	F 5′ TGACACTGGCAAAACAATGCA 3′
	R 5′ GGTCCTTTTCACCAGCAAGCT 3′
EGR1	F 5′ CACCCCAGACCAGAAGC 3′
	R 5′ GCTGGGTTTGATGAGCTG 3′
MyoD	F 5′ GCCGGTGTGCATTCCAA 3′
	R 5′ CACTCCGGAACCCCAACAG 3′
Myogenin	F 5′ GACCTGATGGAGCTGTATGAG 3′
	R 5′ CTGAAGGTGGACAGGAAGG 3′
NDGR1 M/H	F 5′ TGAAATGCTTCCTGGAGTCC 3′
	R 5′ GTGGGACACCACCATGTC 3′
CST6	F 5′ CGAGACACGCACATCATC 3′
	R 5′ CCACAAGGACCTCAAAGTCA 3′
p21 H	F 5′ GGAACTTCGACTTTGTCACC 3′
	R 5′ CAGTGACAGGTCCACATGG 3′
PTEN M/H	F 5′ GCTATGGGATTTCCTGCAG 3′
	R 5′ CTAGCTGTGGTGGGTTATGG 3′
TBX2	F 5′ ATGAGAGAGCCGGCGC 3′
	R 5′ CTTGGGCGACTCCCGG 3′
BAX	F 5′ GCTGCAGAGGATGATTGC 3′
	R 5′ CCTTGAGCACCAGTTTGC 3′

Primers indicated M/H detect both human and mouse transcripts.
